# Determinants of Cesarean Delivery During Wartime in Atbara, Sudan: A Case-Control Study

**DOI:** 10.7759/cureus.77130

**Published:** 2025-01-08

**Authors:** Khalid Nasralla

**Affiliations:** 1 Obstetrics and Gynecology, College of Medicine, Qassim University, Buraydah, SAU

**Keywords:** body mass index: bmi, conflict, parity, still birth, women employment

## Abstract

Background: Maintaining an optimal balance in cesarean delivery rates is important to prevent both overuse and underuse of the procedure. Efforts to optimize cesarean delivery rates should consider sociodemographic and obstetric risk factors.

Materials and methods: A case-control study was conducted on women who gave birth in Atbara Maternity Hospital to assess risk factors associated with cesarean deliveries. A questionnaire and hospital records were used to collect data.

Results: The cesarean delivery rate during the study period was 406 (39.69%). The study participants were 187 in the case group and 187 in the control group. The multiple regression analysis showed that history of previous cesarean delivery (adjusted odds ratio (AOR) = 43.25, 95% confidence interval (CI): 19.11−97.86, p ˂ 0.001) and rural residence (AOR = 1.74, 95% CI: 1.01−2.97, p= 0.045) were important determinants of cesarean delivery. Other factors, including age, parity, body mass index, educational level, consanguinity, displacement, maternal employment, number of antenatal care visits, previous history of miscarriage, and newborn birth weight, gender, and outcome, were not associated with cesarean delivery.

Conclusion: The history of previous cesarean deliveries and rural residences were the main determinants of cesarean deliveries in this study.

## Introduction

Cesarean delivery is defined as fetal delivery through an incision in the abdominal wall and uterus after the age of viability. The first cesarean delivery was performed in 1020 AD, and the procedure has gained popularity since then [1]. When performed for a genuine indication, cesarean delivery is a lifesaving procedure that significantly reduces both maternal and neonatal mortality. However, using unwisely can result in serious consequences [2]. Unnecessary cesarean deliveries may lead to avoidable maternal suffering, inefficient use of health resources, and longer hospital stays [3]. In regions where the prevalence of cesarean delivery is low, the procedures are usually insufficient to address all the life-threatening conditions; conversely, in areas with high cesarean rates, many procedures are performed without genuine life-saving indications [4]. In Switzerland, around 8% of all cesarean deliveries were found to have no medical indication [5], which suggests the overuse of the procedures in some areas without genuine indication, while in other regions, the cesarean delivery rates may not be enough to address all serious cases. This disparity was shown in a study conducted in Sudan, showing different rates of cesarean deliveries across the country [6]. To avoid insufficient utilization or overuse of the procedure, the World Health Organization (WHO) advocated 10 to 15% cesarean deliveries in each country for optimal maternal and neonatal outcomes [7].

Currently, 18 million cesarean deliveries are performed each year all over the globe [8], and it is projected that by the end of this decade, one in every three births will be delivered surgically, with an estimated 38 million procedures per year [9]. A large worldwide study conducted between 1990 and 2018 estimated the global rates of cesarean delivery; they found the lowest records in sub-Saharan Africa (5.0%), while the highest rates were in the Caribbean and Latin America (42.8%) [10]. Recent African records are relatively high and exceed WHO’s recommended rates. The recorded rate in Ethiopia is 39.1% [11], 17.3% in Uganda [12], 44% in Angola [3], 17.6% in Nigeria [13], 36.9% in Kenya [14], and up to 57.2% in rural Egyptian districts [15].

Maternal deaths after cesarean delivery are 100 times more likely in low-income countries compared to high-income countries. Also, in low-income countries, nearly one-third of the babies born by cesarean section die [9]. In fact, 86-87% of maternal deaths and 84% of perinatal deaths worldwide occur in Sub-Saharan Africa and South-East Asia, and despite all efforts performed, maternal and neonatal mortality have plateaued in these poor countries [16-19], where cesarean deliveries either fall below the WHO recommended rate (10-15%) in some countries or are overutilized in others. The fact that maternal and infant mortality rates can be reduced by increasing access to cesarean delivery should be combined with better selection of women for the operation, establishing more healthcare facilities, improving the safety of the procedure, enhancing the knowledge of both patients and providers [20,21], and also improving the prediction of patients at higher risk of cesarean delivery.

Besides the obstetric risk factors of cesarean delivery, many researchers have shown a clear association between demographic, economic, and cultural factors and cesarean delivery rates [12,22-25]. These factors may influence a woman’s preference and ability to engage in the decision-making process regarding the mode of delivery.

In Sudan, a recent nationwide study published in 2022 revealed increasing cesarean delivery rates from 4.3% in 2006 to 6.7% in 2010 and 9.1% in 2014. Additionally, there was considerable variation across the country, with higher rates in the Northern region (7-25%) and lower rates in Darfur (2-3%) [6]. After April 15th, 2023, the date on which the Sudanese war began, the country suffered the largest worldwide internal displacement crisis, with more than 7.1 million people displaced inside the country. Thirteen percent of the internally displaced population targeted the Northern states seeking safety and services. According to the International Organization for Migration, this surge of displaced individuals overwhelmed the public services and resources in the areas of arrival, and almost 80% of the displaced population reported either absent or inadequate health services [26-28]. In addition to the crimes committed during the war, the health system was further crippled by the shortages of medical supplies and personnel [29].

This study aims to investigate the sociodemographic factors associated with cesarean delivery in Atbara Maternity Hospital, River Nile state, in Northern Sudan, during such a challenging situation. The findings of this study may help identify women at a higher risk for cesarean delivery and optimize patients’ selection in such resource-constrained settings. The study may also help garner regional and international attention.

## Materials and methods

Study area

Atbara Maternity Hospital is located in Atbara, one of the most influential cities in the River Nile State of Northern Sudan. It is about 310 kilometers northeast of Khartoum and represents an important industrial and transport hub. Atbara Maternity Hospital is the largest government-run maternity hospital in the River Nile state. It provides services to women from Atbara and nearby cities. The hospital staff provides ante-natal and post-natal clinic services free of charge.

Subjects and study design

This case-control study assesses potential risk factors associated with cesarean deliveries. The study was conducted on women who gave birth at Atbara Maternity Hospital between July 14, 2024, and September 24, 2024. The guidelines of the Strengthening the Reporting of Observational Studies in Epidemiology (STROBE) initiative were strictly followed [30].

Variables

The outcome of this study was the cesarean delivery. The exposures were the obstetric and sociodemographic factors associated with cesarean delivery, including age, parity, consanguinity, body mass index (BMI), residence, displacement, educational level, occupation, antenatal care visits, history of previous cesarean delivery, history of miscarriage, history of stillbirth, newborn gender, newborn weight, and newborn outcome.

Inclusion and exclusion criteria

Sudanese women who gave birth in Atbara Maternity Hospital after 24 weeks gestation during the study period and agreed to participate were enrolled in the study. Women who gave birth before 24 weeks gestation, women who gave birth to multiple gestation or congenitally malformed babies, women with medical disorders, foreign nationals, and those who refused to participate were excluded.

Sample size calculation

The sample size was calculated using the formula for unmatched case-control studies in OpenEpi (Dean AG, Sullivan KM, Soe MM. OpenEpi: Open Source Epidemiologic Statistics for Public Health. www.OpenEpi.com), following standard methods [31]. An odds ratio of 2.0 was anticipated for sociodemographic risk factors associated with cesarean delivery, with an estimated 30% prevalence of these exposures among the control group. These assumptions were based on previous studies [23,32]. Based on these assumptions, a minimum of 142 cases and 142 controls were required to detect a statistically significant association between sociodemographic factors and cesarean delivery, with a significance level of 5% (α) and a power of 80% (1 - β).

Ethical considerations

The study was conducted in accordance with the Human Rights Declaration of Helsinki and received ethical approval from the Administration of Health System and Research, River Nile State, Sudan, on July 8, 2024. Written informed consent was obtained from all the enrolled women.

Data collection

The study was conducted on women who gave birth in Atbara Maternity Hospital between July 14, 2024, and September 24, 2024. Cases were women who gave birth via cesarean delivery (including both emergency and elective procedures). Controls were women who were delivered vaginally. Data were obtained via direct interviews and questionnaires completed by two resident doctors trained in counseling women and data collection. Data collection was carried out daily using consecutive sampling methods. All women who gave birth in Atbara Maternity Hospital during the study period and met the inclusion criteria were invited to participate in the study after delivery and before discharge. Women were typically discharged four to eight hours after vaginal delivery and two to four days after cesarean delivery.

After obtaining informed consent, a structured questionnaire was administered to each woman to collect information on sociodemographic and obstetric characteristics. These included age, parity, consanguinity, residence, displacement, educational level, occupation, antenatal care visits (˂ 4, ≥ 4), history of previous cesarean delivery, history of miscarriage, history of stillbirth, mode of delivery (vaginal or cesarean delivery, including both emergency and elective), newborn gender (male or female), newborn weight, and newborn outcome. The date of the last normal menstrual period was used to determine the gestational age at delivery. Women’s weight and height were measured using the standard procedures, and their BMI was computed. Hospital records were reviewed to determine the total number of vaginal and cesarean deliveries during the study period.

Statistical analysis

SPSS Statistics version 26.0 (IBM Corp. Released 2019. IBM SPSS Statistics for Windows, Version 26.0. Armonk, NY: IBM Corp) was used to analyze the data. Categorized data were expressed as frequencies (%). A Shapiro-Wilk test was used to evaluate the normality of the continuous variables (age, parity, BMI, and neonatal birth weight), which were found to be not normally distributed and were expressed as medians (interquartile ranges “IQR”). Categorized data are expressed as frequencies (%) and were compared between two groups using a χ2 test. Univariate analysis was conducted with the mode of delivery as the dependent variable. Variables with p < 0.05 were used to build up a multiple regression analysis model and rule out confounders. Adjusted odds ratios (AORs) and 95% CIs were calculated as they were applied. A two-sided p-value of < 0.05 was considered statistically significant.

## Results

The total number of deliveries during the study period was 1,023, and 406 (39.69%) were cesarean deliveries. The study participants were 374; 187 of them were in the case group (women who had cesarean deliveries, both elective and emergency) and 187 in the control group (women who delivered vaginally). Among the case group, 135 (72.2%) were elective cesarean deliveries, and 52 (27.8%) were emergency. Compared to the control group, the history of previous cesarean delivery was significantly higher among cases (cases: 117, 62.6% vs. controls: 7, 3.7%, p ˂ 0.001). Most cases live in rural areas compared to the control group (cases: 118, 63.1% vs. controls: 97, 51.9%, p = 0.028). History of intrauterine fetal death was also significantly higher among cases (cases: 21, 11.2% vs. controls: 10, 5.3%, p = 0.043). There was no significant difference between the two groups in age, parity, BMI, educational level, consanguinity, displacement status, maternal employment, number of antenatal care visits, previous history of miscarriage, newborn birth weight, gender, and outcome. The univariate analysis revealed that a history of previous cesarean delivery (OR = 42.98, 95% CI: 19.10−96.72), rural residence (OR = 1.59, 95% CI: 1.05−2.40), and a history of having intrauterine fetal death (OR = 2.24, 95% CI: 1.02−4.90) were associated with cesarean deliveries (Table 1).

**Table 1:** Univariate analysis of factors associated with cesarean deliveries in Atbara, River Nile State, Sudan, 2024 BMI: body mass index, CI: confidence interval, ANC: antenatal care

Variables		Cesarean deliveries N = 187	Controls (vaginal deliveries) N = 187	Odds ratio (95% CI)	p-value
		Median (interquartile range)			
Maternal age, years		26.0 (16.0-43.0)	28 (16.0-42.0)	0.99 (0.96-1.03)	0.662
Parity		2 (1-9)	2 (1-9)	1.02 (0.91-1.14)	0.735
BMI, kg/m^2^		26.95 (16.53−43.39)	27.34 (16.90-38.93)	0.99 (0.94−1.04)	0.692
Birth weight, Kg		3.0 (1.0-4.0)	2.9 (1.0-4.0)	1.15 (0.77−1.73)	0.500
	Frequency (proportion)				
Maternal education status	Less than Secondary	86 (46.0)	83 (44.4)	Reference	0.755
	Secondary or higher	101 (54.0)	104 (55.6)	0.94 (0.62-1.41)	
Consanguinity	No	84 (44.9)	86 (46.0)	Reference	0.835
	Yes	103 (55.1)	101 (54.0)	1.04 (0.70-1.57)	
Residence	Urban	69 (36.9)	90 (48.1)	Reference	0.028
	Rural	118 (63.1)	97 (51.9)	1.59 (1.05-2.40)	
Displacement	No	131 (70.1)	136 (72.7)	Reference	0.567
	Yes	56 (29.9)	51 (27.3)	1.14 (0.73-1.79)	
Maternal employment status	Unemployed	173 (92.5)	177 (94.7)	Reference	0.401
	Employed	14 (7.5)	10 (5.3)	1.43 (0.62-3.31)	
ANC	˂ 4	62 (33.2)	73 (39.0)	Reference	0.237
	≥ 4	125 (66.8)	114 (61.0)	1.29 (0.85-2.00)	
History of cesarean delivery	No	70 (37.4)	180 (96.3)	Reference	˂ 0.001
	Yes	117 (62.6)	7 (3.7)	42.98 (19.10-96.72)	
History of miscarriage	No	142 (75.9)	147 (78.6)	Reference	0.537
	Yes	45 (24.1)	40 (21.4)	1.17 (0.72-1.90)	
History of stillbirth	No	166 (88.8)	177 (94.7)	Reference	0.043
	Yes	21 (11.2)	10 (5.3)	2.24 (1.02-4.90)	
Gender of newborn	Male	97 (51.9)	84 (44.9)	Reference	0.179
	Female	90 (48.1)	103 (55.1)	0.76 (0.50-1.14)	
Newborn outcome	Alive	181 (96.8)	178 (95.2)	Reference	0.432
	Dead	6 (3.2)	9 (4.8)	0.66 (0.23-1.90)	

Table 2 shows the multiple regression analysis results. Both histories of previous cesarean delivery (AOR = 43.25, 95% CI: 19.11−97.86, p ˂ 0.001) and rural residence (AOR = 1.74, 95% CI: 1.01−2.97, p = 0.045) remained associated with cesarean delivery, while the history of stillbirth (AOR = 2.18, 95% CI: 0.83−5.71, p = 0.112) was not.

**Table 2:** Multiple regression analysis of the factors associated with cesarean delivery in Atbara, River Nile State, Sudan, 2024 OR: odds ratio, CI: confidence interval

Variable		Adjusted OR	95% CI	p-value
Residence	Urban	Reference		0.045
	Rural	1.74	1.01−2.97	
History of cesarean delivery	No	Reference		˂ 0.001
	Yes	43.25	19.11−97.86	
History of stillbirth	No	Reference		0.112
	Yes	2.18	0.83−5.71	

## Discussion

This study showed that a history of cesarean delivery is significantly associated with a higher likelihood of repeat cesarean delivery (AOR = 43.25, 95% CI: 19.11−97.86, p ˂ 0.001). This is consistent with the findings of a prospective cohort study of 480 Brazilian women [33] and two other studies conducted in China [34,35]. Although a trial of labor after a previous cesarean delivery is the recommended strategy to decrease the prevalence and complications of repeated cesarean deliveries [36], pursuing this option requires significant resources, clinical experience, and comprehensive counseling to ensure informed decision [37]. The fear of the potential complications in a resource-limited setting, which is further intensified by war, may prompt practitioners to choose cesarean delivery as a safer alternative. Furthermore, vaginal birth after cesarean delivery practiced in low-resource settings poses a danger to both mother and her baby and can also have serious legal and ethical implications [38].

Women living in rural areas have 1.74 times more risk for cesarean delivery compared to those living in urban areas (AOR = 1.74, 95% CI: 1.01−2.97, p = 0.045). This finding aligns with studies from Egypt and Angola [3,15]. Conversely, other studies from Ethiopia and Uganda showed significantly higher odds of cesarean delivery among women living in urban areas [12,22,23,39]. This reflects the complex and multidirectional association between residence and cesarean delivery. The increased likelihood of cesarean deliveries among rural residents can again be attributed to the lack of healthcare facilities, limited access to specialized expertise, and the lack of autonomy in the decision-making process regarding the mode of delivery for women in rural areas [40]. Other sociodemographic variables, such as age, parity, BMI, displacement, consanguinity, educational level, maternal employment, a history of miscarriage or intrauterine stillbirth, antenatal care visits, newborn outcome, newborn gender, and birth weight, were not associated with cesarean delivery in this study.

Another important finding in this study is the 39.69% prevalence of cesarean delivery, which is significantly higher than a previous report in 2022, prior to the onset of the Sudanese conflict (7-25%) [6]. It is also much higher than the records from neighboring countries of Eritrea (10.1%) [41], Uganda (17.3%) [12], Nigeria (17.6) [13], and Somalia (21.6%) [42]. This high rate is similar to records from Ethiopia (39.1%) [11] and Kenya (36.9%) [14] and remains much lower than the records from Angola (44%) [3], Egyptian rural (57.2%), and urban (54.8%) districts [15].

This significant increase in the rate of cesarean deliveries during the Sudanese conflict contradicts the findings from Syria in a study conducted during the Syrian armed conflict, which showed a reduction in the rates of cesarean deliveries from 35% in March 2017 to 23% in July 2020 [43]. This reflects the unpredictable consequences of armed conflicts. Generally, displacement, shortage of resources, lack of medical supplies, and safety issues are likely to result in a reduction in maternal health-seeking behaviors. However, the shortage of skillful healthcare workers and higher levels of stress during wartime may drive many practitioners to choose quicker methods for delivering babies, which may explain the high cesarean delivery rate in this study.

The limitations of this study include several factors that may affect the generalizability of the findings. The study was conducted in a relatively peaceful city due to the challenges associated with data collection in conflict zones. As a single-center study, the results may not represent other regions in the country. Although meticulous auditing was performed, reliance on hospital records and self-reported data may have introduced recall or reporting bias. Furthermore, cultural beliefs and healthcare provider practices require deeper exploration to enhance understanding.

Lastly, the relatively short data collection period may not sufficiently capture seasonal variations or long-term trends in cesarean delivery rates. Future research that addresses these limitations could provide a more comprehensive understanding of the topic.

## Conclusions

The cesarean delivery rate observed in this study is much higher compared to previous local records. This high rate is mainly associated with the history of previous cesarean delivery and rural residence. The pre-existing shortages in healthcare facilities, resources, and skilled practitioners may be further exacerbated by war. This may increase concerns about the possible complications and lead many healthcare providers, especially in rural areas, to opt for cesarean deliveries as a safer alternative. Other sociodemographic factors reported to be associated with cesarean delivery by other researchers had no association in this study. Further research on the practice of vaginal birth after cesarean delivery in Sudan is required, especially during the current conflict. Also, the equitable distribution of health services between rural and urban areas must be evaluated. Restoring peace and raising adequate financial support is of utmost importance in addressing the shortage of healthcare services and minimizing its consequences.
